# Diffuse large B-cell non Hodgkin's lymphoma in a 65-year-old woman presenting with hypopituitarism and recovering after chemotherapy: a case report

**DOI:** 10.1186/1752-1947-5-498

**Published:** 2011-10-04

**Authors:** Manohara Kenchaiah, Steve L Hyer

**Affiliations:** 1 Department of Endocrinology, Epsom & St Helier University Hospitals NHS Trust, Surrey, SM5 1AA, UK

## Abstract

**Introduction:**

Diffuse large B-cell non Hodgkin's lymphoma may involve the pituitary either as a primary central nervous system lymphoma or, more frequently, as metastasis from systemic lymphoma leading to hypopituitarism. A partial recovery of pituitary function after treatment with chemotherapy has previously been described but complete recovery with cessation of all hormone supplements is excessively rare. We report a patient presenting with anterior hypopituitarism with subsequent complete and sustained recovery of pituitary function after successful treatment of the lymphoma.

**Case presentation:**

A 65-year-old Caucasian woman with lethargy, loss of appetite and peripheral edema was found to have anterior hypopituitarism. Magnetic resonance imaging showed no mass lesions in the pituitary although a positron emission tomography scan showed abnormal pituitary activity. An abdominal computed tomography scan revealed multiple intra-abdominal lymph nodes, which on histology proved diagnostic of diffuse large B-cell non Hodgkin's lymphoma. She received six cycles of R-CHOP chemotherapy, after which she achieved a complete metabolic response at all known previous sites of the disease, confirmed by positron emission tomography scanning. Concomitant with the tumor response, there was full recovery of adrenal, thyroid and gonadal axes which has persisted at 10 months follow-up.

**Conclusion:**

Although rare, it is important to recognize lymphomatous infiltration of the pituitary as a potentially reversible cause of hypopituitarism.

## Introduction

Pituitary involvement of lymphoma either at presentation or late in the disease is rare. Patients with metastasis of lymphoma to the pituitary usually present with diabetes insipidus as the posterior lobe of the pituitary (unlike the anterior lobe) is supplied with blood directly from the systemic circulation [1]. However, patients may present with anterior pituitary failure either as a result of tumor extension from the posterior pituitary or as isolated tumor deposits in the anterior pituitary [2]. We now describe a patient presenting to our endocrine department with anterior hypopituitarism which proved fully reversible following successful chemotherapy.

## Case presentation

A 65-year-old Caucasian woman presented with a six-week history of lethargy and loss of appetite. She had also noticed progressive pedal edema for a few weeks. She had no significant past medical history. An initial examination revealed that she was pale and hypotensive (blood pressure 98/60 mmHg) with significant peripheral edema.

Her investigations revealed hyponatremia (118 mmol/L), abnormal liver function tests with albumin 25 g/L, alkaline phosphatase 244 units/L, alanine transaminase 61 units/L, aspartate transaminase 61 units/L, and pancytopenia with hemoglobin 11 g/dL, white blood cell count 3.0 × 10^9^/L, platelet count 99 × 10^9^/L. A 250 μg short tetracosatrin test showed cortisol results at 0 minute of 93 nmol/L, 30 minutes of 43 nmol/L and 60 minutes of 315 mmol/L, suggestive of adrenal failure. Baseline plasma adrenocorticotropic hormone was reduced at 4 ng/L (reference range 10 ng/L to 40 ng/L) in keeping with secondary hypoadrenalism. She was started on hydrocortisone replacement and showed slight improvement. Further endocrine tests showed thyroid-stimulating hormone 0.35 mU/L, free thyroxine 7.1 pmol/L, free tri-iodothyronine less than 1.7 pmol/L, suggesting secondary hypothyroidism; follicle stimulating hormone 6.3 U/L and luteinizing hormone 1.2 U/L, indicating secondary hypogonadism; serum prolactin 343 mIU/L and serum insulin-like growth factor 1 34 nmol/L (normal range 6 nmol/L to 36 nmol/L). There was no evidence of diabetes insipidus. These tests were interpreted as showing generalized anterior pituitary dysfunction. Our patient received thyroxine replacement in addition to hydrocortisone and was subjectively improved. Even after steroids, there was no evidence of diabetes insipidus.

**Figure 1:**
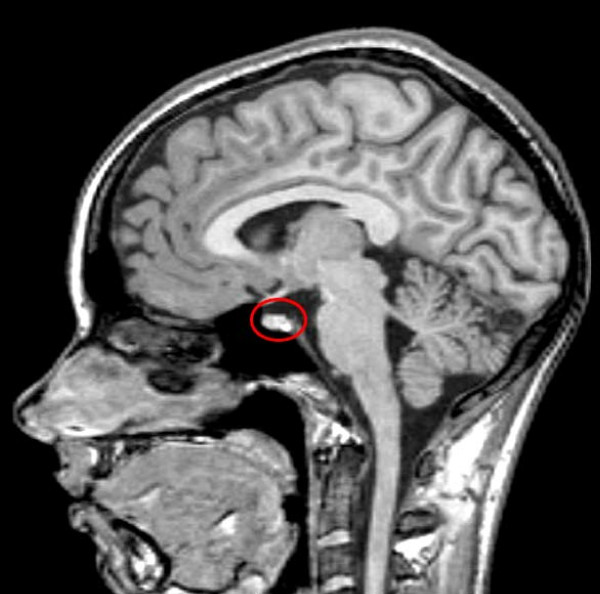
**T1-weighted MRI of her pituitary with contrast showing normal appearances of pituitary (circled in red)**.

**Figure 2:**
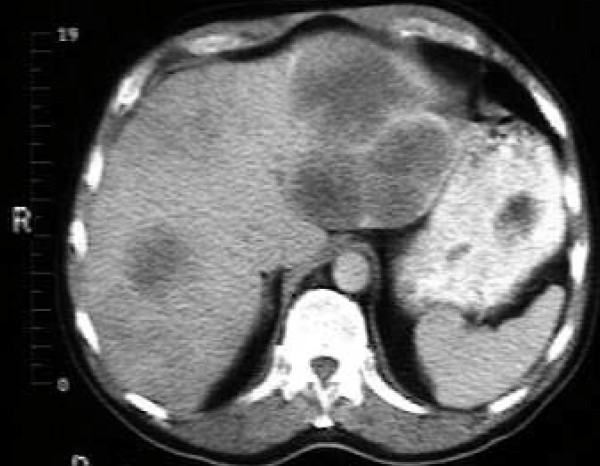
**CT of her abdomen revealing multiple low density opacities in the liver consistent with metastases**.

Magnetic resonance imaging (MRI) of her pituitary (Figure 1) revealed no mass lesions in her pituitary. In view of her peripheral edema, an abdominal and pelvic ultrasound was performed which revealed multiple intra-abdominal lymph nodes, multiple solid liver lesions and a 2 cm right groin lymph node (Figure 2). Full body computed tomography (CT) confirmed the ultrasound findings as well as identifying involvement of the base of her skull. She underwent a biopsy from a right groin lymph node, which showed reactive changes only. Subsequently a liver biopsy was reported as showing a highly malignant anaplastic tumor of unknown origin.

**Figure 3:**
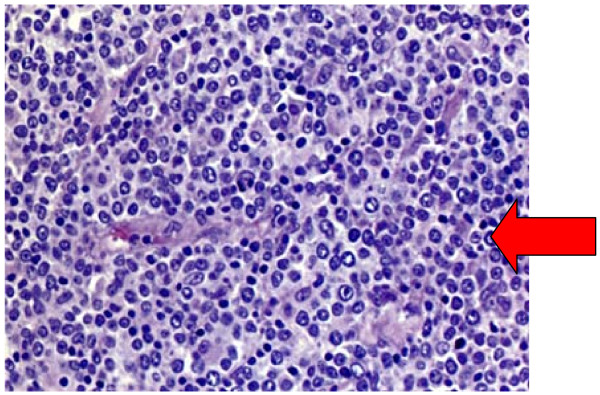
**Liver biopsy revealing infiltrates of large lymphocytes, confirmed as large B-cell non Hodgkin lymphoma on special staining (hematoxylin and eosin, original magnification × 200)**.

**Figure 4:**
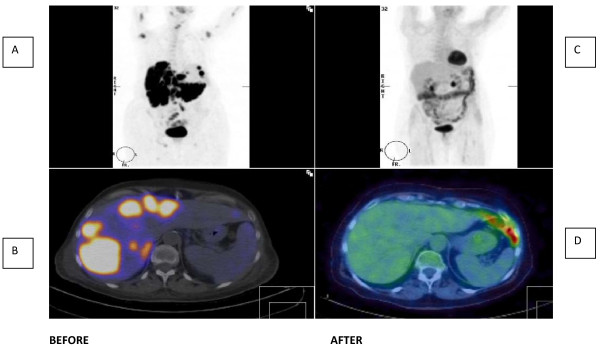
**PET scan imaging before and after six cycles of R-CHOP**. **(A) **PET scan before chemotherapy. Note multiple metabolically active areas in the liver, axillae, neck and base of skull. **(B) **PET-CT scan before chemotherapy demonstrating widespread metastases in liver. **(C) **PET scan after chemotherapy; complete resolution of metastases with only physiological appearances in gut. **(D) **PET-CT scan after chemotherapy showing complete resolution of liver metastases.

Our patient was referred to the local oncology unit where histology was reviewed. Immunophenotyping was performed with CD19, CD20, CD22, CD79 and B-cell lymphoma 2 and the diagnosis was confirmed as diffuse large B-cell non Hodgkin's lymphoma (Figure 3). A positron emission tomography (PET) scan (Figure 4) confirmed widespread disease including the pituitary (Stage 4).

Our patient received six cycles of chemotherapy with rituximab, cyclophosphamide, vincristine, doxorubicin and prednisolone (R-CHOP). Repeat PET scanning showed a complete metabolic response at all known previous sites of the disease (Figure 4).

At the same time, steroid and thyroxine replacement doses were progressively withdrawn under close monitoring. There was full recovery of her adrenal, thyroid and gonadal axes and she was off all medication and clinically well when reviewed in the endocrine clinic 10 months after completing chemotherapy (see Table 1).

**Table 1:** Endocrine investigations before/after R-CHOP chemotherapy

	At presentation	Four months after treatment	Eight months after treatment
**FSH** **(U/L)**	6.3	25.1	38.8

**LH** **(U/L)**	1.2	16.0	25.8

**9 a.m. Cortisol (nmol/L)**	93	412	395

**FT4** **(pmol/L)**	7.1	12.2	14.2

**TSH** **(mU/L)**	0.35	0.5	0.5

LH: luteinizing hormone; FSH: follicle stimulating hormone; FT4: free thyroxine; TSH: thyroid-stimulating hormone

## Discussion

We describe a patient with diffuse large B-cell lymphoma metastasizing to the anterior pituitary, in whom pituitary function was restored after successful treatment of the lymphoma. An early report of complete resolution of non Hodgkin's lymphoma involving the pituitary was based on ^67^gallium scintigraphy and the patient remained on hormone replacement [3]. More recently, partial recovery of anterior hypopituitarism was reported after successful chemotherapy but the patient continued to require thyroxine [4]. Complete and persistent pituitary recovery as in this case report is very rare.

Consistent with previous case reports [5,6], diffuse large B-cell lymphomas are the commonest types of lymphoma to involve the pituitary or central nervous system. Primary pituitary lymphoma is exceedingly rare. They usually present with headache, cranial nerve abnormality and evidence of pituitary insufficiency [7]. By contrast, pituitary metastasis may be asymptomatic presumably because of the pituitary's large reserve capacity. When symptomatic, posterior lobe involvement is more common, but isolated anterior pituitary dysfunction was present in six of a series of 13 patients previously reported [8].

Typically, MRI reveals a pituitary mass which on T1- and T2-weighted images is either iso- or hypodense and enhances with contrast [9]. Unlike other brain tumors, T2-prolongation is not present owing to the dense cellularity and high nucleus to cytoplasm ratio of lymphoma [10]. In patients with posterior pituitary involvement, there is typically absence of the pituitary bright spot. In our patient, the pituitary magnetic resonance image was normal, which we presume indicates a diffuse infiltration by lymphoma cells rather akin to a hypophysitis with infiltrating lymphocytes.

Differential diagnosis of a pituitary mass has been well described previously [2]. In a patient with a normal pituitary image and anterior hypopituitarism, the differential includes other infiltrative processes including sarcoidosis and hypophysitis.

Our patient was not subjected to a pituitary biopsy as we felt we had sufficient evidence of lymphomatous infiltration of the pituitary based on clinical findings, histology from liver and lymph nodes and PET scan results. Pituitary lymphomas are described as firm, vascular and adherent to surrounding structures and needing piecemeal dissection. This is in contrast to necrotic adenomas which are typically soft [9]. In our patient, the simultaneous improvement in pituitary function with tumor response to chemotherapy was also highly suggestive.

Prognosis in previous reported series ranges between nine weeks after presentation to greater than 20 years [5]. Prognostic factors include age, comorbidity and histology at presentation. However widespread lymphoma, as in our patient, is not, in itself, indicative of poor prognosis.

## Conclusion

We report a patient with diffuse large B-cell non Hodgkin's lymphoma with anterior pituitary involvement, whose pituitary function recovered with successful chemotherapy. A complete metabolic response was induced as reflected by the PET scan imaging and no abnormal activity is now present in the pituitary. Unusually, the pituitary was not enlarged on imaging and we speculate that the infiltration behaved like a hypophysitis and completely resolved with chemotherapy. At follow-up, our patient is well and off all medication, 10 months after initiation of chemotherapy. Early diagnosis is important as aggressive disease is potentially rapidly fatal and treatment can be effective.

## Consent

Written informed consent was obtained from the patient for publication of this case report and any accompanying images. A copy of the written consent is available for review by the Editor-in-Chief of this journal.

## Competing interests

The authors declare that they have no competing interests.

## Authors' contributions

MK collected data, performed the literature search and wrote the first draft of the manuscript. SH supervised the project, reviewed literature and revised the manuscript. Both authors read and approved the final manuscript.

## Funding

No funding was received in the preparation of this report
